# Clinical Value of Optical Coherence Tomography Angiography in Neovascular Age-Related Macular Degeneration

**DOI:** 10.3390/jcm15135013

**Published:** 2026-06-27

**Authors:** Samuel Asanad, John Thomspon

**Affiliations:** 1 Department of Ophthalmology, Keck School of Medicine, University of Southern California, Los Angeles, CA 90033, USA; 2 Greater Baltimore Medical Center, Baltimore, MD 21204, USA; jthompson@retinaspec.com

**Keywords:** optical coherence tomography angiography, fluorescein angiography, neovascular age-related macular degeneration, manual resegmentation

## Abstract

**Background/Objectives**: The utility of optical coherence tomography angiography (OCTA) for neovascular age-related macular degeneration (nAMD) remains unclear. The current study investigated the choroidal neovascularization (CNV) detection rate by OCTA in comparison with standard fluorescein angiography (FA) and spectral-domain optical coherence tomography (SD-OCT). **Methods**: Subjects underwent multimodal imaging, including FA, SD-OCT, and OCTA imaging, which were compared. In patients with unilateral nAMD, the contralateral eye with dry AMD (n = 39) was included to determine imaging modality sensitivity and specificity. Eyes with inaccurate automated segmentation from retinal distortion were manually resegmented. **Results**: The diagnostic performance for nAMD was 86% sensitivity and 100% specificity by OCT (AUC: 0.93; 95% CI 0.87–0.99; *p* < 0.001); 82% sensitivity and 100% specificity by FA (AUC: 0.91; 95% CI 0.84–0.98; *p* < 0.001); and 68% sensitivity and 100% specificity by automatically segmented OCTA (AUC: 0.84; 95% CI 0.76–0.93; *p* < 0.001). OCTA diagnostic accuracy improved following manual resegmentation to 88% sensitivity and 100% specificity (AUC: 0.94; 95% CI 0.89–1.0; *p* < 0.001). Diagnostic accuracy of OCT combined with manually resegmented OCTA (AUC: 1.0; 95% CI 1.0–1.0; *p* < 0.001) was greater than that of OCT or FA combined (AUC: 0.96; 95% CI 0.92–1.0; *p* < 0.001) but both were very accurate. **Conclusions**: Manual segmentation of the OCTA images can help identify CNV in eyes otherwise undetected by automated segmentation algorithms due to errors in segmentation of retinal layers. Eyes with substantial elevation in one or more layers of the retina were most likely to benefit from resegmentation.

## 1. Introduction

Early detection and prompt management of neovascular age-related macular degeneration (nAMD) is crucial for the prevention of vision loss [1,2]. Currently, the diagnosis and management of nAMD rely heavily on fluorescein angiography (FA) and spectral-domain optical coherence tomography (SD-OCT). Although these imaging modalities are highly effective, they have some limitations, including the need for invasive intravenous dye injection, which carries a small risk of anaphylaxis and may be contraindicated in patients with renal impairment or allergies [3,4]. The use of OCT alone to make the diagnosis of nAMD also has limitations in patients with AMD [5]. Other causes of intraretinal fluid such as cystoid macular edema, intraretinal clefts overlying geographic atrophy, subretinal fluid in eyes with central serous retinopathy, or non-neovascular pigment epithelial detachment (PED) can be misinterpreted as representing nAMD. In addition, these modalities will not accurately detect certain aspects of CNV lesions, such as their depth and vascular flow characteristics [5,6,7,8,9].

Optical coherence tomography angiography (OCTA) is a non-invasive imaging modality that can provide high-resolution images of retinal and choroidal blood vessels without the need for dye injection. Previous studies using different OCT devices and algorithms have reported the sensitivity and specificity of CNV detection in the range of 50–100% [10,11,12,13,14]. Prior reports have also described various effects of anti-VEGF therapy on the flow signal within CNV, including a reduction in size, complete flow resolution, and persistence of flow [2,15,16,17,18,19,20]. The utility of OCTA in the diagnosis and management of nAMD in treatment-naïve eyes has not been fully explored, and its utility as a complementary or alternative imaging modality to FA and SD-OCT is being evaluated. Therefore, the role of OCTA for choroidal neovascularization (CNV) detection in nAMD is being further refined. The current study investigated the CNV detection rate in treatment-naïve eyes with nAMD by OCTA in comparison with standard FA and SD-OCT.

## 2. Materials and Methods

### 2.1. Study Design

**Figure 1:**
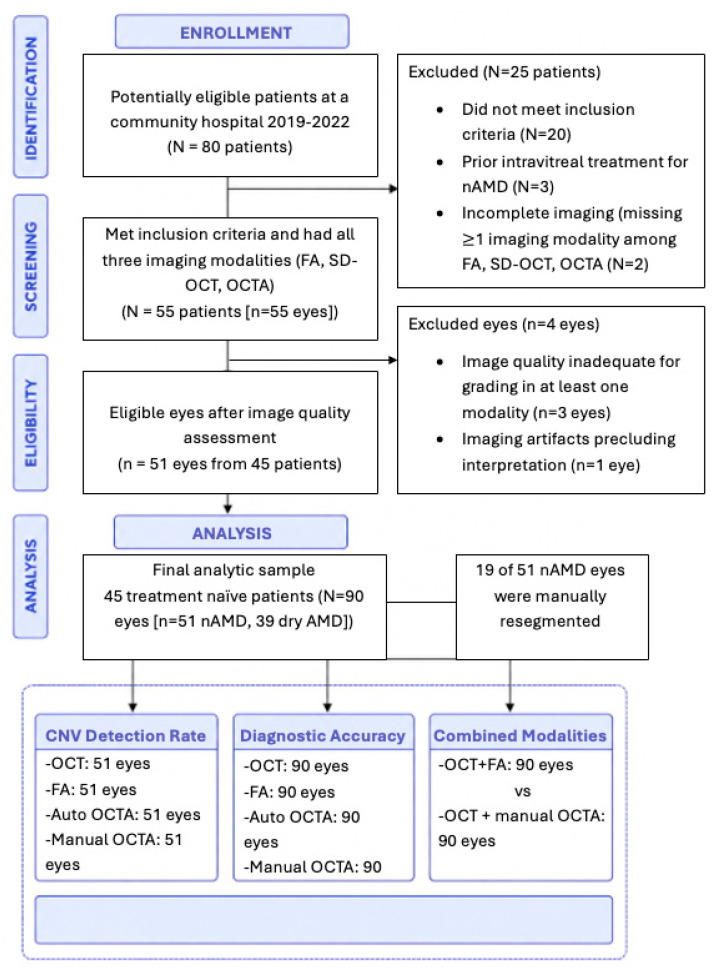
The CONSORT-style flow diagram illustrates patient enrollment, exclusions at each stage, and final analysis.

We conducted a retrospective, cross-sectional study from 2019 to 2022 at a community hospital consisting of 80 potential patients. Eyes with significant media opacity, poor signal strength (Q score below 15), motion artifacts, projection artifacts, co-existent diabetic maculopathy, pathologic myopia, or retinal vein occlusion were excluded. The resulting study comprised 51 eyes from 45 treatment-naïve patients diagnosed with new onset nAMD by two retinal imaging experts using a multimodal imaging protocol. The subjects included in this study were (1) patients over 50 years with clinical features of age-related maculopathy, such as soft or hard drusen and pigmentary alterations and (2) macular exudative signs on at least one of the two imaging examinations (FA or SD-OCT). All patients underwent FA (Zeiss FF4, Carl Zeiss Meditec AG, Oberkochen, Germany or Heidelberg, Heidelberg Engineering, Heidelberg, Baden-Wurttemberg, Germany), SD-OCT (Spectralis Heidelberg, Heidelberg Engineering, Heidelberg, Baden-Wurttemberg, Germany), and OCTA imaging (Spectralis OCTA Module, Spectralis Heidelberg, Heidelberg Engineering, Heidelberg, Baden-Wurttemberg, Germany). All three imaging modalities were available to the experts who established the reference standard. Figure 1 shows the cohort details.

### 2.2. OCTA Image Analysis

All OCTA images were acquired using the SPECTRALIS 2 SD-OCTA (Heidelberg Engineering) with macular scans centered on the fovea. As previously described, the OCTA software (Heyex Software Version 1.9.201.0, Heidelberg Engineering, Heidelberg, Germany) algorithm automatically segments the retinal and choroidal layers [21,22]. The projection artifact removal (PAR) tool utilizes information from the Superficial Vascular Plexus to remove artifacts from OCTA images of the outer retina. The automated, real-time mode combined with the TruTrack Active Eye Tracking System of the SPECTRALIS OCT2 improves the signal-to-noise ratio [21]. Structural OCT section images and the corresponding blood flow information are combined into a fusion image, superimposing the OCTA signal on the structural OCT section image to provide a direct visual correlation of structural and flow information. The SPECTRALIS OCTA en face images are based on the sum of the OCTA signal between defined slab boundaries and a contrast function.

**Figure 2:**
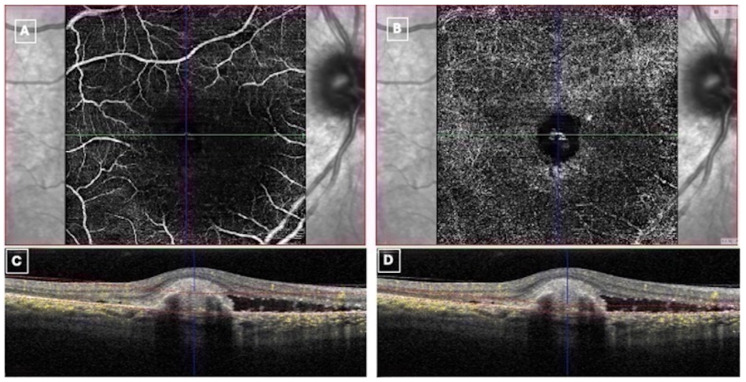
Automated segmentation vs. manual resegmentation en face OCTA (**A**,**B**) with cross-sectional B-scans and flow overlay (**C**,**D**). Distortion of the retinal layers precludes CNV detection on en face OCTA (**A**) by automated segmentation (red dotted slab boundary lines, (**C**)). Manual resegmentation within the fibrovascular PED (red dotted slab boundary lines, (**D**)) shows a vascular hyperintense decorrelation signal corresponding to a neovascular lesion on en face OCTA (**B**).

**Figure 3:**
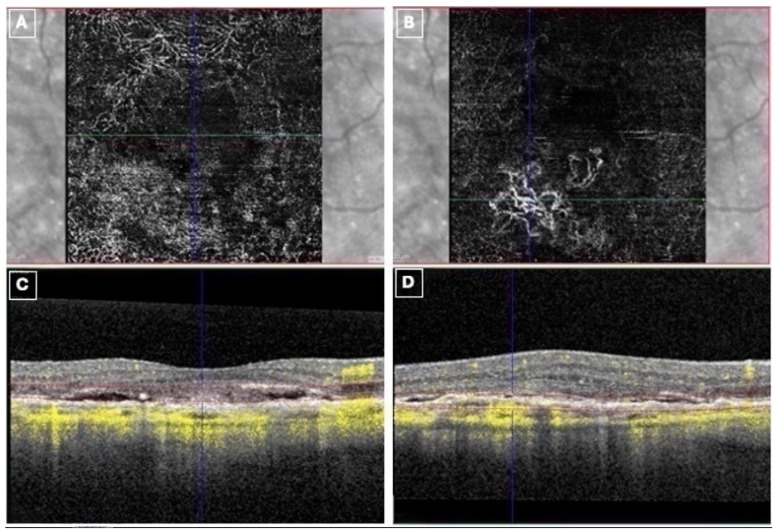
Automated segmentation vs. manual resegmentation en face OCTA (**A**,**B**) with corresponding cross-sectional B-scans and flow overlay (**C**,**D**). Distortion of the retinal layers precludes CNV detection on en face OCTA (**A**) by automated segmentation (red dotted slab boundary lines, (**C**)). Manual resegmentation (red dotted slab boundary lines, (**D**)) shows a CNV lesion on the en face scan (**B**) corresponding with the hyperintense flow signal below the RPE (**D**).

The SPECTRALIS OCTA adaptive retinal slab permits a continuous and interactive display of the structures between the internal limiting membrane (ILM) and basement membrane (BM). This provides a robust visualization of vasculature independent of the disruption of retinal layers caused by pathology. The thickness of the adaptive retinal slab and its location within the retina can also be changed interactively while the upper and lower slab boundaries automatically adapt to the shape of the ILM and BM. The adaptive retinal slab feature enables real-time dynamic scrolling with precise isolation of the strata between the ILM and BM to differentiate between superficial and deep capillary networks. Image evaluation was performed by two masked, experienced graders independently (SA, JT). This study was initiated because the graders did not know which modalities or combination would be most accurate. The quality of the images was reviewed, and poor-quality scans (Q score < 15) were excluded from the analysis. Classification of CNV subtypes was defined based on the presence of a vascular decorrelation signal within the neurosensory retina (CNV Type III), immediately above the RPE (CNV Type II), below the RPE, and/or in between the Bruch’s membrane and the RPE layers (CNV Type I). Manual resegmentation of the retinal layers using the adaptive retinal slab feature was performed in suspicious eyes with undetectable CNV by automated segmentation algorithms due to distortion and misidentification of retinal layers. OCTAs with detectable CNVs were not resegmented as the abnormal vessels were already clearly delineated by the automated algorithm (Figure 2 and Figure 3).

### 2.3. Statistical Analysis

Sensitivity and specificity of OCT, FA, and OCTA were calculated. In patients with unilateral nAMD, the contralateral eye with non-exudative or dry AMD was included for the determination of imaging modality specificity. Cohen’s Kappa coefficient was calculated as a measure of inter-rater reliability between graders. OCTA manual resegmentation was performed only for cases where the automated OCTA did not detect CNV. Grader ratings were compared for manual resegmentation of the “negative automated OCTAs” to assess the increased rate of CNV detection. Mixed-effect models were used to examine the main effect of nAMD diagnosis and eye laterality as a repeated measure. Receiver operating characteristic curve (ROCC) analysis was used to assess the diagnostic ability of the binary classification system as measured by the area under the curve (AUC). The plotted curves represent the ROC performance derived from the predicted probabilities of the logistic regression classifiers rather than threshold-independent smoothed ROC estimations. The line segments shown reflect the discrete operating points produced by the available prediction probabilities across the dataset. To test the differences between the ROC curves, the area under the ROC curves was compared using the Hanley–McNeil method [23]. Statistical significance was assumed at *p* < 0.05. To prevent false positives across multiple comparisons, we applied a Bonferroni correction, dividing the standard α = 0.05 significance level by the four tests performed, thereby requiring an adjusted *p*-value of ≤0.01 to establish statistical significance. Analysis was performed using SPSS V.20 package software.

## 3. Results

Of the 80 patients initially enrolled in the study, 25 were excluded due to either significant media opacity, poor signal strength (Q score below 15), motion artifacts, projection artifacts, co-existent diabetic maculopathy, pathologic myopia, or retinal vein occlusion. We included 45 treatment-naïve patients (mean age: 82.8 ± 7.7 years) comprising 51 eyes with nAMD and 39 eyes with dry AMD (Table 1).

Table 2 shows the classification of CNV by type and the relative detection rates by OCT, FA, and OCTA. Cohen’s Kappa as a measure of inter-rater reliability between graders for manual resegmentation was 0.96 (*p* < 0.001). There was no significant correlation between nAMD diagnosis and eye laterality (*p* = 0.09).

Among the 51 eyes with nAMD, manual resegmentation of the retinal layers was performed for 19 eyes (37%) comprising seven, ten, and two eyes for types I, II, and III CNV, respectively. Total CNV detection rate by OCTA prior to manual correction was 71% as shown in Table 2. Manual correction of OCTA segmentation increased overall CNV detection rate, ranging between 82 and 88% depending on the grader. Type I, II, and III CNV detection rates increased with manual segmentation, ranging between 90 and 100%, 89 and 92%, and 0 and 50%, for type I, II, and III CNV, respectively (Table 2).

### 3.1. Classification of nAMD vs. Dry AMD

Table 3 illustrates the sensitivities and specificities of the imaging modalities.

**Figure 4:**
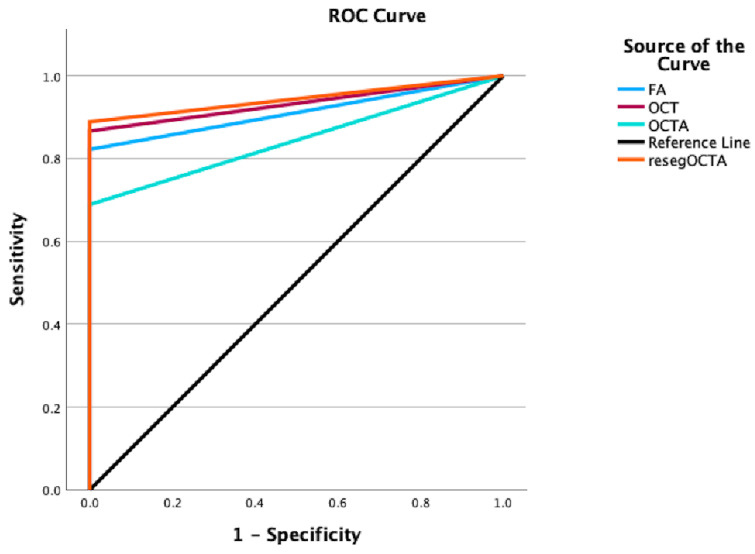
Representation of receiver operator curves (ROCs) of four binary logistic regression analyses to classify nAMD disease classification. The area under the curve (AUC) of FA was 0.91 (blue). The AUC of OCT was 0.93 (red). The AUC of automatically segmented OCTA was 0.84 (teal). The AUC of manually resegmented OCTA was 0.94 (orange).

Logistic regression was used to assess the diagnostic accuracy of OCT, FA, and automated as well as manually resegmented OCTA for classifying nAMD diagnosis (Figure 4).

### 3.2. OCT

The diagnostic performance of OCT in detecting CNV showed 86% sensitivity and 100% specificity (AUC: 0.93; 95% CI 0.87–0.99; *p* < 0.001).

### 3.3. FA

The diagnostic performance of FA in detecting CNV showed 82% sensitivity and 100% specificity (AUC: 0.91; 95% CI 0.84–0.98; *p* < 0.001).

### 3.4. OCTA Automated Segmentation

The diagnostic performance of OCTA in detecting CNV by automated segmentation showed 68% sensitivity and 100% specificity (AUC: 0.84; 95% CI 0.76–0.93; *p* < 0.001).

### 3.5. OCTA Manual Resegmentation

The diagnostic performance of manually resegmented OCTA in detecting CNV showed 88% sensitivity and 100% specificity (AUC: 0.94; 95% CI 0.89–1.0; *p* < 0.001).

### 3.6. Comparative Analysis

**Figure 5:**
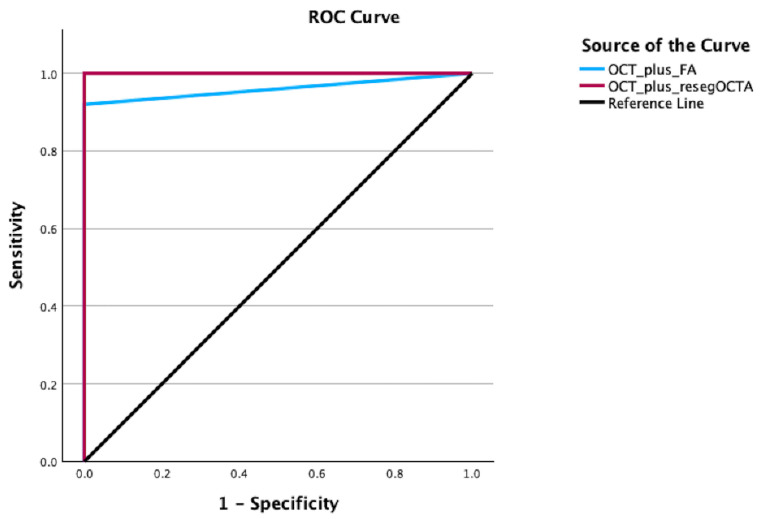
Representation of receiver operator curves (ROCs) of combined imaging modality binary logistic regression analyses to classify nAMD disease classification. The area under the curve (AUC) of OCT plus FA was 0.96 (blue). The AUC of OCT plus manually resegmented OCTA was 1.0 (red).

The area under the curve (AUC) of OCTA by manual resegmentation was significantly greater than that of the automated segmentation algorithm (*p* = 0.02). We further explored a classification model that could maximize discriminatory potential by combining imaging modalities (Figure 5).

The diagnostic performance of OCT in combination with FA for detecting CNV showed 92% sensitivity and 100% specificity (AUC: 0.96; 95% CI 0.92–1.0; *p* < 0.001). The diagnostic performance of OCT in combination with manually resegmented OCTA for detecting CNV showed 100% sensitivity and 100% specificity (AUC: 1.0; 95% CI 1.0–1.0; *p* < 0.001). The AUC of the OCT and manually resegmented OCTA combined model was statistically significantly greater than that of the OCT and FA combined model (*p* < 0.009).

## 4. Discussion

The current study performed a comparative analysis of OCT, FA, and OCTA diagnostic accuracy for nAMD. We evaluated the detection rates of OCT, FA, and OCTA for CNV subtypes I, II, and III exclusively in treatment-naïve eyes with nAMD. We also assessed a classification model for maximizing nAMD diagnosis using combined imaging modalities. OCT combined with manually resegmented OCTA correctly classified the retinal disease class of nAMD vs. dry AMD with high sensitivity and specificity. Taken together, these findings highlight OCTA’s utility as a clinically valuable diagnostic tool for nAMD.

The diagnostic performance of OCTA in nAMD has been previously studied. Notably, however, reported sensitivities for commercially available OCTA systems have been widely variable, ranging between 32 and 100% [10,11,12,13,14,24]. The majority of these studies either included both naïve and treated patients or did not differentiate between CNV lesion types [12,25,26,27,28]. Most previous investigations were also conducted using different imaging platforms, which also have reported limitations [5]. Several researchers have also assessed the performance of swept-source OCTA in nAMD. These groups have similarly deviated in their findings depending on whether the en face scan was used alone or in conjunction with cross-sectional OCTA [5,11,13,14,20,24,29]. Not surprisingly, the diagnostic utility of OCTA in nAMD is variable, suggesting the need for further investigation to more clearly elucidate its clinical value.

In our study, manually resegmented OCTA increased nAMD diagnostic accuracy by OCTA as compared to the built-in automated segmentation algorithm. Diagnostic challenges in CNV detection commonly occur when there is misidentification of the retinal layers from significant distortion of the retinal architecture found in many eyes with nAMD [4,30]. Pigment epithelium detachments can attenuate the OCTA signal, preventing visualization of neovascularization beyond the RPE [4]. The accumulation of intra- and subretinal fluid can cause severe retinal distortion and misidentification of the retinal layers using the automated segmentation algorithm, thus resulting in false-negative CNV detection by OCTA [5]. In our study, approximately 30% of eyes necessitated manual resegmentation. This agrees with other studies which have similarly shown that segmentation errors can occur in up to 50% of eyes evaluated with OCTA [30,31]. We used the combination of en face and cross-sectional B-scan with flow overlay for CNV detection. This enabled a more precise localization of the CNV by providing functional (angiogram) as well as morphologic (OCT B-scan and en face scan) information simultaneously [3,14]. More importantly, we employed the adaptive retinal slab feature to visualize retinal morphology and the vasculature independent of the disruption of retinal layers. This enabled us to perform a complete layer-by-layer analysis of the entire retina to detect vascular indices suggestive of CNV. Decorrelation flow signals distinct from a projection artifact on cross-sectional OCTA were evaluated in detail to determine the presence of a CNV lesion. Manual adjustment of the B-scan from the outer retinal boundaries to the deep choroid also permitted a layer-by-layer, depth-resolved tomographic visualization of the neovascular network. These features likely explain the higher detection rates for type I and type II CNV, which were found to be more difficult to detect by the AngioVue OCTA [4,5,32,33].

Few studies have evaluated the diagnostic performance of the SPECTRALIS OCTA device in nAMD. Coscas et al. reported a global CNV detection sensitivity of 95% for types I, II, and III comprising both treatment-naïve and treated eyes with nAMD. Importantly, however, CNV detection specificity was not determined and the effect of manual resegmentation was not measured [34]. Ansary et al. also compared the diagnostic performance of CNV detection by manual versus automatic OCTA segmentation [5]. They showed a CNV detection sensitivity of 68% by automatic segmentation using this device, which closely resembles ours [5]. In this same study, Ansary and colleagues reported a diagnostic potential AUC of 0.83 by manual segmentation OCTA, whereas our study demonstrated an AUC of 0.94 [5]. Manual resegmentation in our study was performed using the adaptive retinal slab feature, allowing an interactive and complete layer-by-layer analysis of the entire retina. The procedure for manual resegmentation across multiple OCTA devices is not well defined by Ansary et al. in their study [5]. This difference in resegmentation protocol may explain the higher diagnostic potential exhibited by OCTA in our study.

We further evaluated the potential of combined imaging modalities to maximize group classification of nAMD vs. dry AMD. Our analysis showed that OCT in conjunction with manually resegmented OCTA enhanced the diagnostic accuracy of nAMD, exceeding that of OCT combined with FA. OCT provides high-resolution structural images of retinal morphological changes but does not provide an image of the size of the precise location of the neovascular membrane complex itself. Alternatively, FA provides dynamic blood flow information including vascular leakage of active CNV, but lacks structural morphologic data available in the OCT and OCTA [34]. Many FA platforms allow some image manipulation with the FA such as increasing contrast. Retina specialists routinely use the early and late frames of the angiogram to help diagnose choroidal neovascularization while the OCT-A uses a single reconstructed image. The automated segmentation is inaccurate in identifying the appropriate layers in the retina if there is substantial elevation in the macula so the OCT-A resegmentation can be used to help correct these errors. Previous studies have demonstrated good concordance between SPECTRALIS OCTA and traditional multimodal imaging [34]. In our study, OCT and OCTA had similar sensitivity and specificity in CNV detection overall. Our subgroup analysis also showed variability in detection rate as a function of CNV type. Specifically, detection of type I and II CNV was lower by OCT relative to manually resegmented OCTA. Conversely, detection of combined type 1 and II and type III CNV was lower by OCTA relative to OCT as previously reported [29]. Taken together, these findings suggest that OCTA can potentially bridge the gap between imaging modalities by combining the structural information of OCT with some of the vascular anatomy available from the FA.

Limitations of this study include those associated with an exploratory approach. The current study did not perform indocyanine green angiography which may have given additional information about the vascular pattern of the nAMD, especially for type I CNV. We also note that the detection of combined Type I and II and Type III may have been limited by the low number of classified subtypes, which were only 5% for each. To truly comment on the sensitivity and specificity of identifying each lesion type with different imaging modalities, the number of eyes in each subtype should be much larger. The Type I/II combined and Type III subgroups contained only two cases each, so the reported detection rates are not intended to support definitive subtype-specific conclusions and can be influenced by sampling variability. In turn, these subgroup findings are exploratory and should not be overinterpreted. In our analysis, we used a mixed-effects model to account for within-subject correlation, which may not entirely remove the underlying biological clustering of fellow eyes. The specificity estimates are internally derived estimates rather than fully independent observations. External validation in an independent cohort may be warranted.

The increase in CNV detection rate in our report was operator dependent, like the uncertainty and subjectivity in evaluating FA and OCT in some eyes with CNV [4]. Trained operators are necessary to determine when there are projection artifacts. Projection artifacts can usually be identified by comparing the adjacent retinal layers and observing that the vessels are normal retinal or choroidal vessels. We also measured the kappa coefficient to assess interrater reliability. Results reflect performance on the Spectralis platform with the specified software version and the graders/operators involved, and may not fully generalize to other clinical settings, devices, or grader backgrounds. Manual resegmentation depends on a solid understanding of vascular slab classification within the Spectralis OCTA Module software, as would be expected among retina specialists who routinely use OCTA as a diagnostic imaging modality in clinical practice. The associated learning curve may vary according to the user’s OCTA experience and familiarity with clinical interpretation. Manual resegmentation adds only a limited amount of extra time without meaningfully disrupting clinic workflow once the operator is experienced with the process. We acknowledge the exclusion rate and its implications for generalizability. The current study prioritized data quality to clearly demonstrate the utility of manual resegmentation.

This highlights the importance of accurate segmentation for optimal OCTA interpretation. Readers will be able to improve their ability to resegment OCTA to improve detection of nAMD with experience. This study helps to better define the utility of OCTA as a useful clinically tool and its performance using a multimodal imaging approach. These findings will hopefully stimulate additional studies to improve the detection of nAMD using OCTA. Improvements in OCTA resolution and software algorithms should improve the diagnostic accuracy of OCTA without the need for manual resegmentation.

## Figures and Tables

**Table 1:** Cohort demographics and clinical features. Abbreviations: AMD, age-related macular degeneration.

	Neovascular AMD	Dry AMD
No. of Subjects (eyes)	45 (51)	39 (39)
Age, years (SD)	82.8 (7.7)	82.3 (7.7)
Men (%)	18 (40%)	16 (41%)
Women	27 (60%)	23 (59%)

AMD = age-related macular degeneration; SD = standard deviation.

**Table 2:** Detection rate of choroidal neovascularization for the various subtypes by OCT, FA, OCTA, and manually resegmented OCTA. CNV, choroidal neovascularization; OCT, optical coherence tomography; FA, fluorescein angiography; OCTA, optical coherence tomography angiography.

CNV Type	N, Eyes	OCT	FA	OCTA	Resegmented OCTA	
					*Grader 1*	*Grader 2*
I	10	90%	70%	60%	90%	100%
II	37	84%	85%	81%	89%	92%
I and II	2	100%	100%	0%	0%	0%
III	2	100%	100%	0%	0%	50%
Total	51	86%	83%	71%	82%	88%

**Table 3:** Sensitivity and specificity of OCT, FA, automated segmentation OCTA, and manual segmentation OCT in detecting neovascular age-related macular degeneration. *p* values represent the statistical significance of the AUC for a given imaging modality. Abbreviations: OCT, optical coherence tomography; FA, fluorescein angiography; OCTA, optical coherence tomography angiography; AUC, area under the curve.

Imaging Modality	Sensitivity (%)	Specificity (%)	AUC	*p*
OCT	86	100	0.93	<0.001
FA	82	100	0.91	<0.001
Automated OCTA	68	100	0.84	<0.001
Manual OCTA	88	100	0.94	<0.001

## Data Availability

The original contributions presented in this study are included in the article. Further inquiries can be directed to the corresponding author.
